# IMLADS: Intelligent Maintenance and Lightweight Anomaly Detection System for Internet of Things

**DOI:** 10.3390/s19040958

**Published:** 2019-02-24

**Authors:** Tao Qin, Bo Wang, Ruoya Chen, Zunying Qin, Lei Wang

**Affiliations:** 1 MOE KLINNS Lab, Xi’an Jiaotong University, Xi’an 710049, China; nyf8848@stu.xjtu.edu.cn (B.W.); yaer0812@stu.xjtu.edu.cn (R.C.); qzying@mail.xjtu.edu.cn (Z.Q.); wangshuaige@stu.xjtu.edu.cn (L.W.); 2 Shenzhen Research School, Xi’an Jiaotong University, Shenzhen 518057, China; 3 Network Information Center, Xi’an Jiaotong University, Xi’an 710049, China

**Keywords:** Internet of Things, system security monitoring, intelligent maintenance, lightweight anomaly detection, mobile agent

## Abstract

System security monitoring has become more and more difficult with the ever-growing complexity and dynamicity of the Internet of Things (IoT). In this paper, we develop an Intelligent Maintenance and Lightweight Anomaly Detection System (IMLADS) for efficient security management of the IoT. Firstly, unlike the traditional system use static agents, we employ the mobile agent to perform data collection and analysis, which can automatically transfer to other nodes according to the pre-set monitoring task. The mobility is handled by the mobile agent running platform, which is irrelevant with the node or its operation system. Combined with this technology, we can greatly reduce the number of agents running in the system while increasing the system stability and scalability. Secondly, we design different methods for node level and system level security monitoring. For the node level security monitoring, we develop a lightweight data collection and analysis method which only occupy little local computing resources. For the system level security monitoring, we proposed a parameter calculation method based on sketch, whose computational complexity is constant and irrelevant with the system scale. Finally, we design agents to perform suitable response policies for system maintenance and abnormal behavior control based on the anomaly mining results. The experimental results based on the platform constructed show that the proposed method has lower computational complexity and higher detection accuracy. For the node level monitoring, the time complexity is reduced by 50% with high detection accuracy. For the system level monitoring, the time complexity is about 1 s for parameter calculation in a middle scale IoT network.

## 1. Introduction

With the increasing scale and complexity of the Internet of Things (IoT), the security monitoring task of the IoT has become more and more difficult. Firstly, the number of nodes in the IoT system is huge and most of them are heterogeneous [1]. They use different operation systems, have different computing capabilities and different security management policies, which pose greatly challenges for IoT stable running and security monitoring. In general, we can keep the system under control by clearing the vulnerabilities through patching, and by reducing the anomaly influence based on suitable anomaly detection and control policies. But those methods are difficult to implement in IoT. Because the IoT today contains thousands of heterogeneous nodes, the administrators cannot clear all the vulnerabilities by patching with their limited time and energy. Secondly, most of the traditional abnormal detection methods occupy too much local computing resources. To solve these challenges, we developed an intelligent maintenance and lightweight anomaly detection system for IoT. In the newly designed system, not only is the detection performance improved, but the time complexity is also reduced.

Firstly, we employ mobile agents to design the monitoring system and reduce the computational complexity. A mobile agent is a combination of artificial intelligence technology, computer network technology and distributed technology [2]. It can automatically transfer to other nodes to perform the pre-set security monitoring task after it finishes the status checking task of one specific node. The mobility is handled by the mobile agent running platform. The mobile agent can transfer to any nodes installed in the running platform. The mobility is irrelevant with the operation systems only, and only if the nodes are installed in the mobile agent running platform. The detailed migration path is designed by the administrator. This new computing mechanism can effectively reduce the network load, computational complexity, and improve the system stability and scalability [3]. The mobile agents perform data collection and analysis using the local computing resource, which is different from the traditional static agents and can greatly reduce the amount of data transferred from the terminal nodes to the control server. Furthermore, when the system is expanded, we only need to design a new mobile agent to perform the new tasks. The mobile agents are independent with each other and when one mobile agent loses its function, it does not affect other mobile agents. In this paper, we adopted the IBM Aglet platform [4] for system design and mobile agent running. 

We install the mobile agent running platform to some of the nodes in the LAB network. We also developed mobile agents according to the principle of the running platform to perform security monitoring. To evaluate the performance of the developed methods, we deploy one popular botnet captured by the botnet detection system set in our LAB to one of the nodes to generate node level abnormal behavior patterns. We simulate a DDoS attack to generate system level abnormal behavior patterns. Those abnormal behavior patterns are employed to evaluate the performance of the developed methods.

We divided the monitoring tasks into the node level and system level according to the data used. For the node level security monitoring, we designed a new method to reduce the computational complexity from two aspects: the process of data collection and the process of anomaly detection. Firstly, we design a regular interval waiting method to reduce the data collection frequency, which means the data collected is the representative information of a certain time window. Therefore, the amount of data collected is reduced while the computational complexity for the subsequent algorithm is reduced. Secondly, we adopt the Principal Components Analysis (PCA) to extract the main features from the data collected to reduce the data dimensions. And we employ the Density-based Spatial Clustering of Applications with Noise (DBSCAN) clustering method to roughly separate the data collected into normal instances and possible anomalies. In general, the normal points are clustered into clusters with lots of points, and abnormal points are clustered into clusters with less points or isolated as outliers. As a single outlier cannot accurately determine whether the node is in abnormal status or not, we employ the continuous sliding time window to improve the detection accuracy. 

For the system level security monitoring, we employ the number of packets used for communication between different nodes as a parameter to perform anomaly detection. We employ the sketch to calculate the parameter. The sketch is a kind of probabilistic dimension reduction technique, whose computational complexity is constant and suitable for parameter calculation in a large scale network. The items of the sketch at different time points reflect the user’s behavior and the dynamic changing trends. As the user’s behavior has a kind of inertia, they will not change sharply within a short time period, which indicates that the selected parameter will not change sharply in a short time period, which can be employed to perform abnormal detection. In this paper, we employ the EWMA (Exponentially Weighted Moving-Average) method to forecast the dynamic changing trends, and select the confident up and low bounds as thresholds to perform anomaly detection. 

Finally, we design different agents to perform the response policies to control the time of the anomalies and reduce their influence and mitigate the risks. The response strategies are classified into different categories according to the characteristics of the anomalies detected. The first kind is the system maintenance agents. This kind of mobile agent transfers to the specific node and performs a patch to reduce the virus infection probability. Those agents only transfer to the specific node which holds vulnerabilities. The second one is those which can perform Anti-Virus. They transfer to the specific node that infected the virus. The third one is the quarantine management mobile agent, it transfers to the special node and performs different quarantine policies to control the abnormal behavior while does not affect the normal behavior heavily.

In conclusion, the main contribution of this paper is summarized as follows:

1. The mobile agent is cross-platform, it can transfer to any of the nodes that are installed on the mobile agent running platform. Most importantly, this new computing mechanism can not only reduce the number of data collection agents, but also greatly reduce the amount of data transferred from the terminal nodes to the control server.
2. We design lightweight anomaly mining methods for the node level and system level security monitoring. The computational complexity of node level security monitoring method is reduced by combining several methods, and that of the system level is constant and irrelevant with the system scale.

The rest of this paper is organized as follows: Section 2 presents a simple lecture review. Section 3 presents the introduction of the Intelligent Maintenance and Lightweight Anomaly Detection System (IMLADS). Section 4 presents the extracted features and the anomaly mining methods designed. Performance evaluation results are given in Section 5. Conclusion and future work then follow in Section 6.

## 2. Related Works

This paper mainly focuses on developing an intelligent maintenance and lightweight anomaly detection system for IoT. The related work mainly includes the abnormal detection methods and the intrusion detection systems, we simply summarize the related works as follows:

### 2.1. Anomaly Detection Methods

Abnormal detection methods build the users’ normal profiles from their historical activities and mark the ones which deviate from the normal profiles as possible anomalies. There are many kinds of data that can be used to build the normal behavior profiles. The authors in Ref. [5] discussed how to use the key stroke biologic characteristics to build the profiles and perform identity authentication. They verified that different users have different key stroke habits. The authors in Ref. [6] employed the mouse data to perform user identification and detect abnormal behaviors. They presented a feasible approach to extract the main behavior characteristics from the mouse data and found it is useful for real-time and continuous identity authentication. By monitoring the characteristics of the node level log, some special attacks such as adding backdoors or inserting Trojan horses to the system can be detected [7,8]. The authors in Refs. [9,10] proposed an abnormal detection model using the subset of the system calls, they verified that system calls can reflect a user’s behavior characteristics and can be used for abnormal behavior detection. The authors in Refs. [11,12] selected five types of features, including RAM usage, node network connections and usage of bandwidth etc., to perform anomaly detection. The experimental results verified by their method have good performance in node level anomaly detection. To select suitable features for abnormal detection, an intelligent algorithm with feature selection and decision rules is proposed in Ref. [13], in which SVM is used to extract the most important features. Although those methods have high detection accuracy, their deployment scale is limited by the data collection mechanism.

### 2.2. Abnormal Detection System

The abnormal detection system is usually called the Intrusion Detection System (IDS), which is simply classified into the HIDS (host based intrusion detection system) and NIDS (network based intrusion system) according to the data used. James Anderson firstly proposed the concept of IDS in 1980, they mainly wanted to help the administrators review the audit trails easily by providing an automatic tool [14]. Along with the development of network and IoT, there are many new security issues [15], and many researchers begin to design and develop IDS suitable for large scale networks and IoT [16,17]. But along with the development of system complexity, it is more and more difficult to develop the real time anomaly detection system due to computational complexity. To face this challenge, researchers employed the FPGA to accelerate the calculation and improve the detection efficiency for online applications [18,19]. Although the intrusion detection system has been developed for many years and there are many applications, there are still some open problems to be solved. Firstly, the data collection and analysis methods usually occupy too much local computing resources, which pose great challenges for real-time monitoring in a large scale IoT system. Secondly, most of the abnormal detection methods used in the IDS are based on signature matching or machine learning, it is very difficult for the administrator to find enough labeled data to train the model. Thus, there are lots of false alarms, which greatly reduce their practicability in the actual network environments.

Enlightened by the related works, we develop an Intelligent Maintenance and Lightweight Anomaly Detection System (IMLADS) for IoT system security management. In the newly designed system, we employ the mobile agent to perform data collection and analysis, which can greatly reduce the computational complexity. In addition, we develop lightweight anomaly detection methods to further reduce the computational complexity.

## 3. Simple Introduction to IMLADS

### 3.1. Introduction to Mobile Agent

The mobile agent is a segment of a program or a soft sensor, which can migrate from one node to another node autonomously in the heterogeneous network and interacts with the nodes on behalf of the administrator to perform pre-set tasks. A mobile agent has the following characteristics [2]:

1. Autonomy: The most important characteristic of a mobile agent is that it can independently complete the pre-set tasks on behalf of the administrator without external interference.
2. Mobility: The mobile agent is not only for a specific node, it can transfer to another node to perform a pre-set task. The mobility is handled by the mobile agent running platform and irrelevant with the node.
3. Adaptability: The mobile agent can change its running state according to the changes of the end node’s environment.
4. Cross-platform: A mobile agent has cross-platform characteristics. A mobile agent running platform provides middleware between agents and the node. A mobile agent can theoretically run on nodes with any operation system.

### 3.2. Introduction to IMLADS

**Figure 1:**
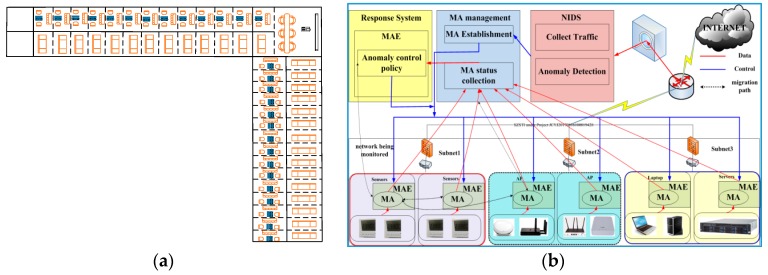
Introduction to Intelligent Maintenance and Lightweight Anomaly Detection System (IMLADS): (**a**) architecture of the building; (**b**) framework of IMLADS.

We designed a system named IMLADS to manage the security issues of our LAB network. The network of our LAB can be treated as a typical IoT network. Figure 1 illustrates the architecture of the building and the framework of IMLADS. In total there are about 40 rooms in our LAB with different kind of end nodes, such as the sensors for temperature and PM 2.5 measurement, laptops, computers, wireless access points and other devices related to research and daily lives. Those nodes distribute in different rooms, the operation system used includes Windows 7, Windows 10, Windows server 2012, and Redhat Linux. They connected with each other through a network and the architecture of the building is shown in Figure 1a. To keep our LAB network running normally and under control, we designed a system named IMLADS and its framework is shown in Figure 1b. We firstly installed the mobile running platform on some of the nodes, then the mobile agent can transfer to any nodes installed on the running platform. The mobility is handled by the running platform and irrelevant to the operation systems. We employ the mobile agents to perform data collection and analysis, after finishing the task at one node, the mobile agent transfers to another node to perform the pre-set tasks. The detailed migration path is designed by the administrator. This running mechanism can greatly reduce the number of data collection and analysis agents in the monitored system. The data is collected and analyzed using the local computing resource, which is different with the traditional centralization system. We also designed lightweight anomaly detection methods for the node level and system level security monitoring. According to the anomalies detected, we design suitable response policies to reduce their influence. If one specific anomaly is reported, the corresponding response policy will be applied automatically by the response agents in turn, keeping the network under control.

### 3.3. Operation Procedure of the IMLADS.

**Figure 2:**
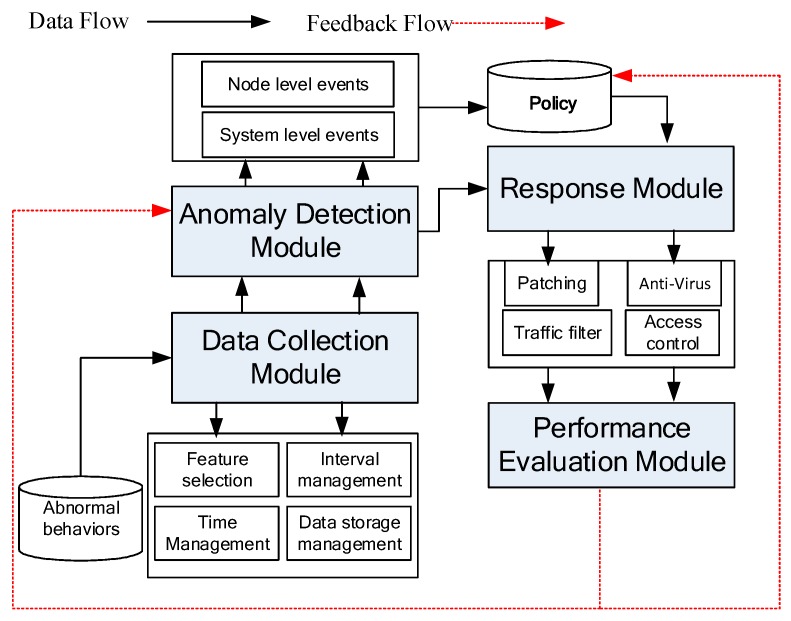
Operation procedure of IMLADS.

The newly developed system mainly contains four modules as shown in Figure 2. The first module is the data collection module, it manages the data collection policy and process. The second one is the anomaly detection module. It includes node level abnormal event detection and system level abnormal event detection. The third one is the response module, it selects a suitable response policy for the anomaly detected. The forth one is the performance evaluation module. It evaluates whether the select policy can control the abnormal behavior and reduce its influence or not. Their relationship is shown in Figure 2, where the black arrow indicates the data flow and the red arrow indicates the feedback flow.

### 3.4. Advantages of IMLADS

According to the characteristics of mobile agents, the designed IMLADS has the following advantages:

1. Reduce network load. Mobile agents can package the administrator’s requests and dispatch them to the destination nodes, and then they perform the designed task using the local computing resource. There is no raw data transferred among the nodes. The mobile agent only reports the anomaly detection results to the control server. If there is no anomaly detected, the mobile agents do not report and transfer to another node selected from the migration path to perform the pre-set task. As the end nodes are working in normal status most of the time, there are only several alarm records transferred from the node to the control server, thus we can greatly reduce the amount of data transferred.
2. Overcome network delay. Real-time or online anomaly detection is very important for network control and delay is unacceptable. The mobile agents transfer to the node directly to perform the tasks designed by the administrators, which is different than the traditional methods, where the node sends its data to the control server and waits for the analysis results. The mobile agent can greatly reduce network latency.
3. Asynchronous and automatic execution. In a heterogeneous network, the nodes contact with each other through the network. But the network service is not stable enough, especially for the wireless nodes, which rely on expensive and fragile network connections. The mobile agents can overcome those shortcomings. They embed the code of a specific monitoring task and once the mobile agents are dispatched, they can operate asynchronously, automatically and independently.
4. Heterogeneity. The mobility is handled by the mobile running platform and irrelevant with the operation systems. The designed system using mobile agents is suitable for performing security monitoring tasks in the heterogeneous IoT network.
5. Robustness and fault-tolerance. Every running mobile agent can transfer to the other end node automatically and independently. If one of the mobile agents loses its efficiency, it will not affect other mobile agents. Thus, the system designed is more robust than that adopting static agents.

## 4. Feature Calculation and Analysis for Security Monitoring

In this section, we introduce the features selected to perform node level and system level security monitoring. We also present the designed lightweight data analysis methods for the node level and system level anomaly mining.

### 4.1. Node Level Feature Selection and Collection

#### 4.1.1. Node Level Selected Features

The appropriate feature is important for the efficiency abnormal detection. In this paper, we choose features including CPU usage, disk read and write, memory usage etc. which are shown in Table 1. These eight features are selected based on the analysis and measurement results of abnormal patterns [20]. For example, an external intrusion always leads to significant changes in disk read and write, CPU or memory usages, and the virus always generate many illegal processes, or more opened ports. 

#### 4.1.2. Collection Method Based on Regular Interval Waiting

During the data collection, agents occupy too much local computing resource due to its frequent queries, resulting in a decline of the local system’s performance. In this paper, we design a data collection method based on a regular interval waiting mechanism to reduce their influence on the system performance. The detailed process is listed as follows:

**Step** **1:** Set waiting time interval Δ*t* and the collection window deadline *t*_0_.
**Step** **2:** Obtain the current system time *t*_1_, IP address, MAC and other features (such as CPU, memory usage, disk read and write, packet flow, process and port).
**Step** **3:** Store the data obtained in Step 2 for further analysis.
**Step** **4:** If *t*_1_ > *t*_0_, then stop the collection process, otherwise increase the time interval Δ*t*, then turn to Step 2. In this way, we can reduce the data collection frequency.

### 4.2. Node Level Anomaly Mining

We focus on developing a lightweight anomaly detection method which occupies the local computing resource as little as possible. Here we combined several technologies to reduce the computation complexity of node level data analysis. 

#### 4.2.1. Data Dimension Reduction Analysis

A high dimension of the collected data causes difficulties for the subsequent clustering algorithm, so we adopt the PCA method to extract the main features and reduce the data dimension [21]. PCA is often used to reduce the data dimension while maintaining the main data characteristics. The detailed steps of PCA are listed as follows:

**Step 1:** Data standardization. Construct the n-dimensional random vectors $x=\left(x_{1},x_{2},x_{3},\ldots ,x_{n}\right)$, where $x_{i}$ represents the data collected from the i-th node. The data is standardized using Equation (1):

(1) $$Z_{\mathrm{ij}}=\frac{x_{\mathrm{ij}}-\bar{x_{j}}}{s_{j}},\;i=1,2,\ldots ,n;\;j=1,2,\ldots ,p$$

where $x_{\mathrm{ij}}$ represents the j-th feature of the i-th node, $\bar{x_{j}}$ represents the average value of j-th feature, $s_{j}$ represents the variance of the j-th feature. The $\bar{x_{j}}$ and $s_{j}$ can be obtained using the following equations:

(2) $$\bar{x_{j}}=\frac{\sum_{i=1}^{n}x_{\mathrm{ij}}}{n}$$

(3) $$s_{j}{}^{2}=\frac{\sum_{i=1}^{n}{\left(x_{\mathrm{ij}}-\bar{x_{j}}\right)}^{2}}{n-1}$$

**Step 2**: Calculate the correlation coefficient matrix R of the standardized matrix Z.

(4) $$R={\left[r_{\mathrm{ij}}\right]}_{p\times p}=\frac{Z^{T}Z}{n-1}$$

where $r_{\mathrm{ij}}$ represents the value of the i-th row and the j-th column of the correlation coefficient matrix R, $Z^{T}$ represents the transpose matrix of standardized matrix Z obtained in Step 1.

**Step 3**: Calculate the eigenvalue using |R−λI|=0. Where R represents the correlation coefficient matrix obtained in Step 2, *I* represents the unit matrix. We select $\frac{\sum_{j=1}^{m}\lambda_{j}}{\sum_{j=1}^{p}\lambda_{j}}>0.7$ as the threshold to select m. Then the eigenvector $b_{i}$ can be obtained using $\mathrm{Rb}=\lambda_{i}b,\;i=1,2,\ldots ,m$.

**Step 4**: Obtain the principal components using Equation (5).

(5) $$U_{\mathrm{ij}}=Z_{i}{}^{T}b_{j},\;i=1,2,\ldots ,n;\;j=1,2,\ldots ,m$$

where $b_{j}$ represents the value of the j-th eigenvector, $U_{\mathrm{ij}}$ represents the i-th node’s data projection on the j-th principal component. 

#### 4.2.2. Abnormal Candidates Detection using the DBSCAN method

After dimensionality reduction, we employ the DBSCAN method to classify the data into different clusters. DBSCAN is a density-based cluster algorithm: given a set of points, it classifies the points which are close to each other into one cluster [22]. In this paper, we use the DBSCAN method to cluster the data into different clusters, the points in the same cluster are close with each other, and they represent similar behavior patterns. As the nodes running under normal status have similar behavior patterns and the anomalies always change the patterns, the normal behavior patterns are classified into one cluster and the abnormal behaviors are classified into another cluster. Thus, the clustering results can be used for abnormal detection. The detailed process of the DBSCAN algorithm is listed in Algorithm 1.

**Algorithm 1.** DBSCAN Clustering Process1. Begin 2. Input: 3.  *U* = {*p*_1_,*p*_2_,…*p*_n_}, *MinPoints*, 4.      Output: 5. *C*_1_,*C*_2_,…*C*_k_//clusters descended by number of elements 6.  *M* = {*m*_1_,*m*_2_,…*m*_n_}//set of noises 7.  k = 0, l = 0 8.  *for ( i = 1; i<= n; i ++)* 9.    *if*
$\sum 1_{\left\{q\in \Omega |dist(q,p_{i})<\varepsilon \right\}}\geq \mathrm{MinPoints}$ 10.  k = k + 1 11.  *else* 12.  if $\sum 1_{\left\{q\in \Omega |dist(q,p_{i})<\varepsilon \right\}}\geq \mathrm{MinPoints}$ 13.      *l* = *l*+1 14.      $m_{k}=p_{i}$ 15. end

We assume C_i_ represents the cluster obtained by the DBSCAN method, and m_j_ represents the outliers. We sort C_1_, C_2_,…C_l_ and m_1_, m_2_,…m_n_ in descending order according to the number of data in the specific cluster, that is, each outlier is also regarded as a single cluster. Thus, all the clusters satisfy Equation (6):

(6) $$\left|C_{1}\right|\geq \left|C_{2}\right|\geq \ldots \geq \left|C_{k}\right|\geq \left|\left\{m_{1}\right\}\right|\geq \left|\left\{m_{2}\right\}\right|\geq \ldots \geq \left|\left\{m_{n}\right\}\right|$$

Based on the actual network management experience, most points represent normal behavior patterns and are classified into one clusters. Thus the number of points in normal clusters and that of abnormal clusters are obviously different to each other. The clusters that represent the normal behavior patterns are much bigger than those of the abnormal behaviors. Therefore, we can conclude that Equation (7), where α represents the ratio of the normal points to the total points, and β represents the points in abnormal cluster, is much less than the points in the normal cluster. We can classify the clusters into different categories and obtain the abnormal point candidates using Equation (7).

(7) $$\{\begin{array}{c} \frac{\left|C_{1}\right|+\left|C_{2}\right|+\ldots +\left|C_{l}\right|}{\sum \left|C_{i}\right|+\sum \left|\left\{m_{1}\right\}\right|}\geq \alpha \\ \frac{\left|C_{l}\right|}{\left|C_{l+1}\right|}\geq \beta \end{array}$$

#### 4.2.3. Detection Accuracy Improvement Based on Continuous Sliding Time Window

**Figure 3:**
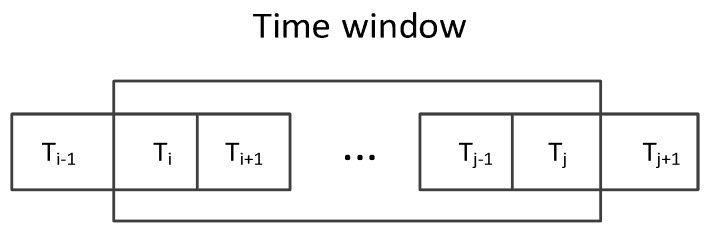
Detection Based on Sliding Time Window.

Beside the virus or attack which can change the node’s profiles, normal operations can also cause sudden changes to the running parameters. Thus a single outlier cannot accurately determine whether the node is in an abnormal status or not, so we cannot simply use the DBSCAN algorithm and regard the outliers as abnormal behavior points. When the node is infected by a virus or under attack, there will be persistent anomaly behavior patterns that last more than one time point. Thus we present an anomaly detection accuracy improvement algorithm based on a continuous sliding time window, as shown in Figure 3, where $T_{i}$ represents the time point.

Within the time window $\Delta t=t_{j}-t_{i}+1$, we use Equation (8) to judge whether an abnormal event is detected. Where ω represents the number of abnormal points in Δt time window. If ω exceeds a certain threshold θ, we regard that there is an anomaly detected, then $A_{\left(t\right)}$ is set to 1, otherwise $A_{\left(t\right)}$ is set to 0. The threshold θ can be selected according to the experimental results. This method based on a continuous sliding time window can avoid the influence caused by a single abnormal point.

(8) $$\{\begin{array}{c} \omega =\sum_{t-\Delta t}^{t}1_{\left\{\mathrm{DBSCAN}\left(t_{i}\right)=-1\right\}}\\ A_{\left(t\right)}=\{\begin{array}{c} 1,\;\omega \left(t\right)\geq \theta \\ 0,\;\omega \left(t\right)<\theta \end{array} \end{array}$$

### 4.3. System-Level Feature Calculation and Anomaly Mining

#### 4.3.1. System Level Features

The traffic packet is the basic information carrier for communication among different nodes, we employ the statistical characteristics of the traffic packets to perform the system level security monitoring, which has been widely used in the network based intrusion detection system [23,24]. The features that can be used include the number of packets, number of bytes or number of flows in a specific time window. The basic assumption of this kind of method is that the user’s normal behavior is stable and will not change sharply in a short time period, but when the abnormal behavior appeared, those statistical features will change sharply. We can employ this kind of sharp change to perform abnormal detection.

In this paper, we employ the total number of packets of a specific node in a specific time window as the feature to perform abnormal behavior detection. We employ the IP address to identify different nodes and do not consider the dynamic host configuration protocol (DHCP)situation. 

#### 4.3.2. Method for Efficiency System Level Feature Calculation

To perform an efficiency system level feature calculation, we employ the sketch to calculate the total number of packets in a specific time window. The sketch method has been widely used for network measurements in the past several years, such as find heavy hitters, heavy changes and estimating flow size distributions. It can also be used for network security monitoring combined with other related methods [25,26,27,28]. 

**Figure 4:**
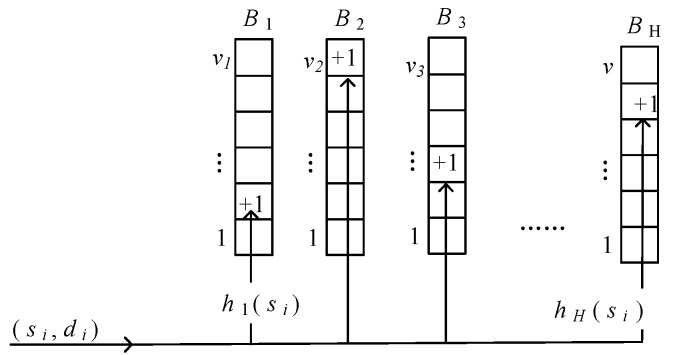
Sketch update procedure.

In this paper, we employ sketch to calculate the defined feature for real-time and lightweight monitoring. The sketch is denoted as *B =* (*B*_1_, …, *B_H_*), where *B_i_* (1 ≤ *i* ≤ *H*) is the hash functions and *H* is the number of different hash functions. *B_i_*[*j*] (0 ≤ *j* ≤ *v*) associate with a hash function *h_i_*: {0, 1, …, *n* − 1}→{0, 1, …, *m_i_* − 1}, where *n* is the size of the space of IP address and *m_i_* is the length of hash functions. The update process of sketch is shown in Figure 4. Initially, the bits in each *B_i_* (1 ≤ *i* ≤ *H*) are all set to zero. When a packet *p_i_* = (*s_i_*, *d_i_*) arrives, each *B_i_* is updated by setting the bit in its column *h_k_*(*s_i_*) according to Equation (9).

(9) $$T[i][h_{k}(s_{i})]=T[i][h_{k}(s_{i})]+1,\;1\leq i\leq H$$

In the sketch, each item of the hash table is corresponding to one specific IP address. The value of the item is the statistical feature of the specific IP address. By analyzing the dynamic changing trends of the value at different time windows, we can achieve the goal of behavior dynamics measurement and system security monitoring.

After calculating the appropriate system level parameters, we need to use the appropriate method to detect whether there are running anomalies. Research shows that the Exponentially Weighted Moving Average (EWMA) control charts show excellent predictive performance under various conditions [29], which can accurately predict and estimate the dynamic changing trends of specific statistical characteristics. We can also use the EWMA control charts to select the up and low bounds to perform anomaly detection. The abnormal behavior will generate a sudden change of statistical characteristic, and the obtained statistical features will exceed the interval determined by the up and low bounds. We employ this mechanism to perform abnormal behavior detection.

#### 4.3.3. Simple introduction to EWMA

For a statistical series X(*n*), EWMA control charts is shown in Equation (10):

(10) $$Ex_{i}=\lambda x_{i-1}+\left(1-\lambda \right)Ex_{i-1}$$

where λ is the forgetting factor, $Ex_{i}$ is the forecasting value of $x_{i}$, the bigger λ is, the more effect of the recent actual value on the forecasting value. We can denote the forecast error of the model by $e_{i}=x_{i-1}-Ex_{i-1}$, based on the forecast error, we can calculate the up and low confidence bounds use Equation (11):

(11) $$\{\begin{array}{c} LCL_{x}\left(i\right)=Ex_{i-1}-L\sigma_{e}\left(i-1\right)\\ UCL_{x}\left(i\right)=Ex_{i-1}+L\sigma_{e}\left(i-1\right)\\ \sigma_{e}^{2}\left(i\right)=\lambda_{1}e{\left(i\right)}^{2}+\left(1-\lambda_{1}\right)\sigma_{e}^{2}\left(i-1\right) \end{array}$$

where $0<\lambda_{1}<1$, it denotes that we can use the EWMA methods to obtain a more stable forecast error. We can select L=1.96 in Equation (11) to obtain a 97% confident interval, which means about 97% of the actual value should be in the confidence interval. If the observed value is out of the interval, we claim that they are caused by anomalies.

## 5. Experiments and Performance Evaluations

### 5.1. Performance Evaluation for Node Level Abnormal Detection Method

In our LAB network, we select 10 nodes as examples and collect their data to verify the efficiency of the developed methods. We also deployed one popular botnet captured by the botnet monitoring system set in our LAB to generate the abnormal behavior patterns [30]. We randomly collected 20 datasets during the system running, each set contained more than 1000 elements, the normal data accounted for 98%, and the rest were abnormal elements. 

#### 5.1.1. Dimension Reduction Efficiency Analysis

We employed the PCA algorithm to the data collected and obtained the correlation coefficient matrix and its eigenvalues shown as follows:

E=[5.622 1.020 0.244 0.101 0.013 0.000]

Based on the eigenvalues obtained, we can obtain the coefficient matrix, which is shown as follows:

$$\mathrm{EigenMatrix}=\left[\begin{array}{ccccccc} 0.000 & -0.000 & -0.007 & -0.145 & 0.020 & -0.989 & -0.004\\ 0.000 & -0.001 & 0.854 & 0.303 & -0.041 & -0.053 & 0.417\\ -0.000 & 0.001 & -0.465 & 0.691 & -0.365 & -0.107 & 0.401\\ 0.000 & -0.020 & -0.008 & -0.591 & -0.705 & 0.071 & 0.386\\ 0.000 & -0.811 & -0.137 & -0.134 & 0.366 & 0.026 & 0.414\\ 0.707 & 0.414 & -0.133 & -0.146 & 0.342 & 0.027 & 0.415\\ -0.707 & 0.414 & -0.133 & -0.146 & 0.342 & 0.027 & 0.415 \end{array}\right]$$

The contribution rates of the each eigenvalue are shown in the Table 2. From the table, we can see that the contribution rates of the first two largest eigenvalues are up to 94.9%, thus we can reduce the data dimension to 2 while the main features of the dataset are still preserved.

We set the *MinPoints* equals to 6 for DBSCAN, the *Eps* is estimated from the *MinPoints* (the minimum value of the average distance between the core object and *MinPoints* nearest neighbors). The experimental results of DBSCAN are shown in Table 3, which indicates that we can still get the consistent cluster results if the original dataset is reduced to an appropriate dimension.

**Figure 5:**
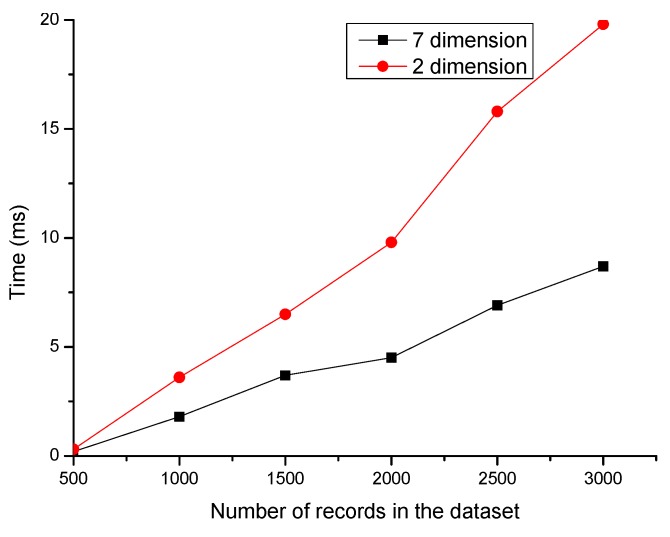
The Relationship between Complexity and Data Dimension.

Figure 5 displays the relationship between computational complexity and data dimension. The red line and the black line represent the clustering execution time of the original seven dimensional data and two dimension data in different datasets. It shows that the method is more efficient after dimensionality reduction. When the dataset contains 1000 elements, the efficiency is improved by 128.8%. Combined with the information in Table 2 and Table 3, and Figure 5, we can conclude that the two-dimensions could effectively reduce the computational complexity of the clustering while preserving the features of the original dataset.

#### 5.1.2. Abnormal Candidates Detection Use DBSCAN

**Figure 6:**
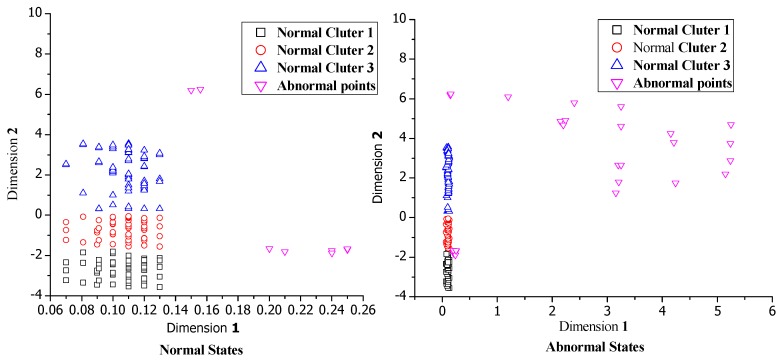
The clustering results of different types of data.

We implement the cluster method to the data after dimensionality reduction. Figure 6 shows the comparison of clustering results between the normal dataset and mixed dataset. The normal dataset is formed by the normal behavior pattern records and the mixed dataset contains some of the abnormal behavior patterns generated by botnet. As the figure shows, the normal state has two kinds of patterns: (1) Silent pattern: there is no user use the system. (2) Normal behavior pattern: the system under normal operations. When the virus infects the node, the clustering results will be changed. The abnormal points gather in small clusters, or isolate as outliers. From the results we can find that the β has little impact on detection results, so we set β to be the number of points in the smallest cluster/(the maximum of the abnormal points in normal dataset + the number of most possible abnormal points), which is the most appropriate value for β. After calculation, we set β to be 5. However, α is more important for the detection accuracy. Based on multi-experimental results, we select α as the ratio of the total elements in the first two largest clusters to the total number of elements in the original dataset. We set α equal to 0.95 based on the definition and calculation.

In order to ensure real-time detection, the sliding window size is set to 10, so the range of ω in Equation (9) is 0≤ω≤10. We can obtain different experimental results with a different threshold θ, and the analysis results are shown in Table 4. We use the detection rate, false positive rate and false negative rate, which are widely used in the anomaly detection areas, to select suitable thresholds, their definitions are given in Equations (12)–(14).

(12) $$\mathrm{Detection}\;\mathrm{Rate}=\frac{\mathrm{TN}}{\mathrm{TP}+\mathrm{TN}}$$

(13) $$\mathrm{False}\;\mathrm{Positive}\;\mathrm{Rate}=\frac{\mathrm{FN}}{\mathrm{TP}+\mathrm{TN}}$$

(14) $$\mathrm{False}\;\mathrm{Negative}\;\mathrm{Rate}=\frac{\mathrm{FP}}{\mathrm{FP}+\mathrm{TP}}$$

where True Negatives (TN) denote the number of the normal events that are correctly classified as normal events. False Positives (FP) denote the number of normal events that are wrongly classified as anomalies, and False Negative (FN) represents the number of anomalies that are wrongly classified as normal events.

With the increase of threshold θ, the false positive rate decreases, but the false negative rate increases sharply. In practical experience, we firstly prefer to reduce the false positive rate. According to the results in Table 4, we can see that when the threshold is between [3, 4], the false positive rate is reduced while the detection rate is up to 95%, so we select the threshold θ equal to 3.

We employ the methods based on signature matching and the methods only use clustering to evaluate the performance of our methods. We select the anti-virus software as an example to represent the methods based on signature matching. In this paper we select the Avast, a commonly and widely used anti-virus software, to evaluate our methods [31]. We deploy the botnet selected from the botnet monitoring system set in our LAB to one of the nodes. We run the botnet in different time periods to generate the abnormal behavior patterns. In total we collected 20 datasets contained in the abnormal behavior patterns and employed them to evaluate our method. The evaluation results are shown in Table 5. From Table 5 we can get that Avast’s performance is disappointed, because it cannot detect unknown anomaly events. It needs to update its signature library frequently to detect newly appeared anomalies. But the botnet used in this paper is captured based on its behavior characteristics. Its signature is not in Avast’s signature library. Thus, Avast cannot detect the botnet and has poor performance. The evaluation results show that our method outperforms the methods based on signature matching. 

In addition, we also compared the false positive rate and false negative rate with the method only using DBSCAN. The results are shown in Table 6. They show that the IMLADS has better performance compared with the method only using DBSCAN, the false positive rate has a great reduction.

### 5.2. Performance Evaluation for Network Level Abnormal Detection Method 

#### 5.2.1. Performance Evaluation of EWMA Method

**Figure 7:**
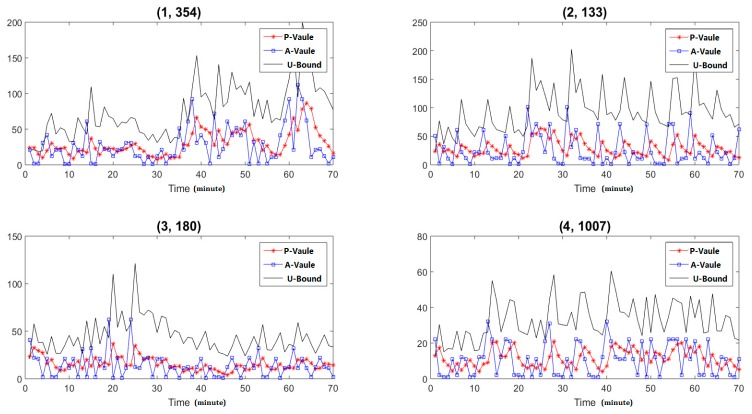
Results of different end nodes.

In sketch, each item of a specific hash function can be tread as the statistical features of each IP address, if there are new packets for a specific node, we need to update its corresponding value of the specific hash function. With time dynamic changing, we obtain the feature dynamic changing trends of the specific IP address by analyzing the specific item values at different time points. Firstly, we select four IP addresses to analyze their feature dynamic changing trends, and the results are shown in Figure 7. In the figure, the item (1, 354) means the item 354 of the first hash function. The P-value means the forecast values of the statistical features, the A-value means the actual of the statistical features, and the U-bound means the up bound of the confident interval. The *X*-axis is the time points and the interval is one minute, the *Y*-axis is the value of the statistical features. From the results we can find that the user’s behavior is dynamic changing and different IP addresses have different dynamic changing trends. The EWMA methods can well forecast the dynamic changing trends.

**Figure 8:**
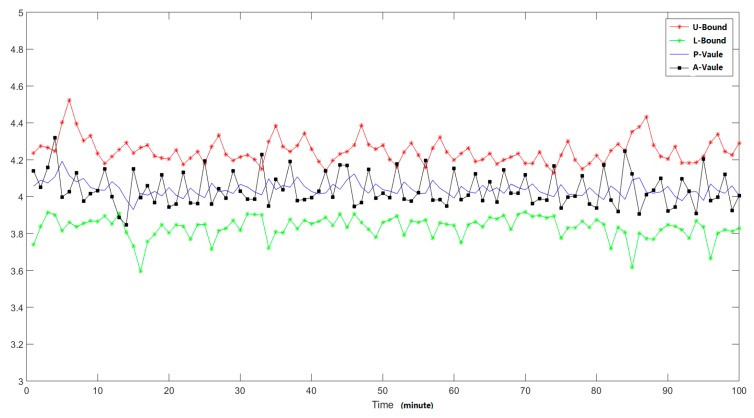
Feature changing trends of all the nodes.

To verify the efficiency of the proposed methods, we injected some abnormal traffic patterns (we simulate the behavior patterns of the DDoS attack with different strength, in this way we can generate the traffic patterns include different abnormal behavior proportions) into the raw traffic captured at different time points. Here we totally injected the abnormal traffic packets at three different time points and the source IP addresses are 125.39.156.* (the last segments is anonymized), the detailed injection time points are the 14th, 31st, and 50th monitoring time points. Firstly, we analyzed the total number of traffic packets of the total end nodes, and the analysis results are shown in Figure 8, where the L-value means the low bound of the confident interval. From the analysis results we can find that there are no abnormal changing trends in the total number of packets, which means there are too many normal traffic packets, and when there are some small number of abnormal traffic packets, we cannot find them through analyzing the features of all the nodes.

**Figure 9:**
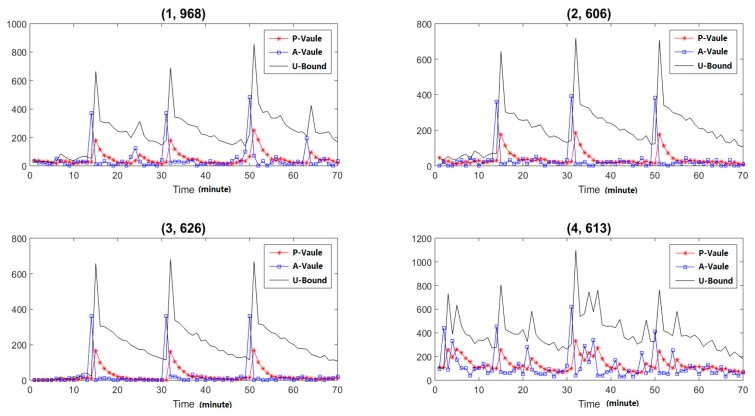
Changing trends of abnormal items.

To detect those slight anomalies, we must analyze the dynamic changing trends of specific nodes, or each item of the hash function. After analysis, we can find there are some abnormalities at the mentioned three time points in (1, 968), (2, 606), (3, 626) and (4, 613), where the first number means different hash functions and the second means the detailed items. The analysis results are shown in Figure 9. As the figure shows, the proposed methods can detect the slight abnormal behaviors. We can also analyze the characteristics of the hash function and obtain the corresponding IP addresses which caused the anomalies for abnormal control.

#### 5.2.2. Method Sensitivity Analysis

As mentioned above, our methods can be used to detect the anomalies that only cause slight abnormal changes in the traffic patterns. We analyze the sensitivity of the proposed methods, which is defined as the number of abnormal IP addresses detected by the algorithm/the total number of abnormal IP addresses. The sensitivity is bigger and the partition of the abnormal IP addresses is bigger, which indicates that the corresponding method only can detect the anomalies after their wider propagation. If the proposed method can achieve a better performance with a small sensitivity, it can find the abnormal behaviors at their early ages. We can control the abnormal behaviors and reduce their influence on the system’s normal running. The detailed analysis results are shown in Table 7. As the table shows, the developed methods can achieve a detection accuracy up to 91.5% with 0.5% sensitivity. Therefore, the proposed method is very effective for low-sensitivity abnormal detection, and can detect the abnormal behaviors at their early age, which is very important for system security management.

### 5.3. Response Policy Design

To control the anomalies efficiently, we divided the anomalies into different categories based on their characteristics, and then we design different agents to perform the response and control policies for different kinds of abnormal behavior. 

Firstly, for the system intelligent maintenance, we design a mobile agent that is responsible for vulnerability patching. This mobile agent transfers among different end nodes. When it finds a specific end node has a vulnerability, it connects to the server to query the patches and perform patching to reduce the system threats. The second mobile agents are Anti-Virus agents. They transfer to specific nodes that are infected with a virus and remove the virus from the node. 

For the anomaly detected, we employ the dynamic quarantine method to remove the threats under the principle of “assume guilty before proven innocent” [32]. We control the abnormal behavior of the nodes by a soft dynamic quarantine method: the quarantine on a node is released after a fixed quarantine time window, such as 120 s. After that, this node can be quarantined again if it is classified into anomalies again. In order to not interfere with the normal activities too heavily, the quarantine on a node is released automatically after a selected time window, even if it has not been inspected by security mangers. 

Finally, for some important end nodes in the monitored system, if they are classified as anomalies, we perform firm quarantine directly and inform the administrator to check their running status immediately. In this way, we can keep the important parts of the system in normal status. 

## 6. Conclusions

This paper proposed an IMLADS for security monitoring of IoT. We employ mobile agents to design the monitoring system, which can greatly reduce the computational complexity and improve system stability and scalability. We also design lightweight anomaly detection methods that can perform anomaly mining with limited local computing resources. For the node level security monitoring, we employ PCA to reduce the data dimensionality, and then employ the clustering and sliding time window model to mine the anomalies. For system level security monitoring, we employ the sketch method to calculate the statistical features quickly. The complexity of the method using sketch is constant and irrelevant with the system scale. We employ the EWMA method to perform dynamic changing trends analysis and the experimental results verify that the developed methods can detect slight anomalies. We also design different agents to perform response policies for different kind of anomalies. The response agents designed cannot only perform system maintenance, but also control the abnormal behavior efficiently. The experimental results based on the platform constructed verify the efficiency and accuracy of the developed methods.

## Figures and Tables

**Table 1:** Node-layer Features.

No.	Features	Simply Descriptions
1	CPU usage	CPU resources occupied by running programs
2	Memory usage	The memory occupied by processes
3	Disk read	The bytes disk read from memory
4	Disk write	The byte written into disk
5	Received packets	The data byte transferred from remote server to the local node
6	Sent packets	The data byte transferred from local node to the remote server
7	Process	The list of running processes in the current time window
8	Port	The list of open ports in the current time window

**Table 2:** The Contribution rates of The Eigenvalues.

Eigenvalues	Contribution Rates	Cumulative Contribution Rates
5.622	80.3%	80.3%
1.020	14.6%	94.9%
0.244	3.5%	98.4%
0.101	1.4%	99.8%
0.013	0.2%	100%
0.000	0.0	100%

**Table 3:** The efficiency of Principal Components Analysis (PCA) Method.

Data Type	# of Clusters	# of ABNORMAL Points	Percentage of Difference
Original data	2	11	0.0%
Three dimension	2	11	0.0%
Two dimension	2	11	0.0%
One dimension	2	1	1%

**Table 4:** Results of different threshold.

Threshold	Detection Rate	False Positive Rate	False Negative Rate
1	98.5%	3.2%	1.5%
2	97.2%	1.0%	2.8%
3	95.5%	0.7%	4.5%
4	94.1%	0.0%	5.9%
5	91.4%	0.0%	8.6%
6	86.5%	0.0%	13.5%
7	77.9%	0.0%	22.1%
8	62.1%	0.0%	37.9%
9	39.8%	0.0%	60.2%
10	14.6%	0.0%	85.4%

**Table 5:** The comparison between IMLADS and Avast.

Algorithm	Detection Rate
IMLADS	95.5%
Avast	0.0%

**Table 6:** The comparison between IMLADS and DBSCAN.

Algorithm	False Positive Rate	False Negative Rate
IMLADS	4.5%	0.7%
DBSCAN	3.1%	42.9%

**Table 7:** Detection results of the different Sensitivities.

No.	Sensitivity Level	Efficiency
1	0.1%	76.8%
2	0.3%	88.2%
3	0.5%	91.5%
4	1.0%	93.8%
5	3.0%	94.8%
6	5.0%	94.9%

## References

[1] Li S., Xu L.D., Zhao S. (2015). The Internet of Things: A Survey.

[2] Mihailescu P. MAE: A Mobile Agent Environment for Resource Limited Devices. https://www.monash.edu/library/researchers/repository.

[3] Zulkarnain Z.A., Hanapi Z.M., Subramaniam S. (2017). A Spawn Mobile Agent Itinerary Planning Approach for Energy-Efficient Data Gathering in Wireless Sensor Networks. Sensors.

[4] IBM Aglets Software Development Kit—Home Page. http://web.media.mit.edu/~stefanm/ibm/AgletsHomePage/index_new4.html.

[5] Obaidat S., Venkata P., Saritha V. (2019). Advances in Key Stroke Dynamics-Based Security Schemes. Biometric-Based Physical and Cybersecurity Systems.

[6] Cai Z., Shen C., Guan X. (2014). Mitigating behavioral variability for mouse dynamics: A dimensionality-reduction-based approach. IEEE Trans. Hum. Mach. Syst..

[7] Liu Z., Qin T., Guan X., Jiang H., Wang C. (2018). An Integrated Method for Anomaly Detection from Massive System Logs. IEEE Access.

[8] Wang Z., Zhu Y. A centralized HIDS framework for private cloud. Proceedings of the 2017 18th IEEE/ACIS International Conference on Software Engineering, Artificial Intelligence, Networking and Parallel/Distributed Computing (SNPD).

[9] Maske S.A., Parvat T.J. Advanced anomaly intrusion detection technique for host based system using system call patterns. Proceedings of the International Conference on Inventive Computation Technologies.

[10] Anandapriya M., Lakshmanan B. Anomaly based host intrusion detection system using semantic based system call patterns. Proceedings of the 2015 IEEE 9th International Conference on Intelligent Systems and Control (ISCO).

[11] Marteau P.F. (2019). Sequence Covering for Efficient Host-Based Intrusion Detection. IEEE Trans. Inf. Forensics Secur..

[12] Gao C., Li Z. Discovering host anomalies in multi-source information. Proceedings of the International Conference on Multimedia Information Networking and Security (MINES’09).

[13] Lin S.W., Ying K.C., Lee C.Y. (2012). An intelligent algorithm with feature selection and decision rules applied to anomaly intrusion detection. Appl. Soft Comput..

[14] Anderson J.P., James P. (1980). Computer Security Threat Monitoring and Surveillance.

[15] Li S., Tryfonas T., Li H. (2016). The Internet of Things: A security point of view. Internet Res..

[16] Elhadj B., Thomas W., Walaa H. (2018). A Critical Review of Practices and Challenges in Intrusion Detection Systems for IoT: Towards Universal and Resilient Systems. IEEE Commun. Surv. Tutor..

[17] Zarpelão B.B., Miani R.S., Kawakani C.T., de Alvarenga S.C. (2017). A survey of intrusion detection in Internet of Things. J. Netw. Comput. Appl..

[18] Viegas E., Santin A.O., Franca A. (2017). Towards an energy-efficient anomaly-based intrusion detection engine for embedded systems. IEEE Trans. Comput..

[19] De França A.L., Jasinski R.P., Pedroni V.A., Santin A.O. Moving network protection from software to hardware: An energy efficiency analysis. Proceedings of the 2014 IEEE Computer Society Annual Symposium on VLSI.

[20] Qiao H., Peng J., Feng C., Rozenblit J.W. (2007). Behavior Analysis-Based Learning Framework for Host Level Intrusion Detection. Proceedings of the IEEE International Conference & Workshops on the Engineering of Computer-Based Systems.

[21] Jolliffe I. (1986). Principal Component Analysis.

[22] Ester M., Kriegel H.P., Xu X. A density-based algorithm for discovering clusters a density-based algorithm for discovering clusters in large spatial databases with noise. Proceedings of the International Conference on Knowledge Discovery & Data Mining.

[23] Giorgi G., Narduzzi C. (2008). Detection of anomalous behaviors in networks from traffic measurements. IEEE Trans. Instrum. Meas..

[24] Garg A., Maheshwari P. PHAD: Packet header anomaly detection. Proceedings of the International Conference on Intelligent Systems & Control.

[25] Schweller R., Li Z., Chen Y. (2007). Reversible sketches: Enabling monitoring and analysis over high-speed data streams. IEEE/ACM Trans. Netw..

[26] Cormode G., Muthukrishnan S. (2004). An improved data stream summary: The count-min sketch and its applications. Latin American Symposium on Theoretical Informatic.

[27] Huang Q., Lee P. LD-Sketch: A distributed sketching design for accurate and scalable anomaly detection in network data streams. Proceedings of the 2014 IEEE INFOCOM.

[28] Callegari C., Giordano S., Pagano M. On the combined use of sketches and CUSUM for Anomaly Detection. Proceedings of the 2015 International Conference on Computing and Network Communications.

[29] Bakdi A., Kouadri A., Bensmail A. (2017). Fault detection and diagnosis in a cement rotary kiln using PCA with EWMA-based adaptive threshold monitoring scheme. Control Eng. Pract..

[30] XJTU, Botnet Detection System. http://botwarden.xjtu.edu.cn.

[31] Avast. https://www.avast.com/index.2016.10.

[32] Zou C.C., Gong W., Towsley D. Worm propagation modeling and analysis under dynamic quarantine defense. Proceedings of the 2003 ACM Workshop on Rapid Malcode.

